# Impact of Social Media News Overload on Social Media News Avoidance and Filtering: Moderating Effect of Media Literacy

**DOI:** 10.3389/fpsyg.2022.862626

**Published:** 2022-03-10

**Authors:** Qiuxia Tian

**Affiliations:** Teaching and Research Section of Communication, Zhujiang College, South China Agricultural University, Gunagzhou, China

**Keywords:** social media news overload, news avoidance, news filtering, need for news, media literacy

## Abstract

In the present era of information technology, people tend to seek out news to enhance their current knowledge and awareness and to gain literacy. The reliance on seeking out news and relevant information has become very necessary to accomplish personal and organizational objectives. The present study has undertaken an inquiry to investigate the impact of social media news overload on news avoidance and news filtering with the mediating and moderating mechanisms of the need for news and media literacy, respectively. For this purpose, data were obtained from 358 Chinese social media users through the aid of survey forms. The data obtained were then analyzed through Smart-PLS software. The statistical technique used for analysis is structural equation modeling (SEM) to determine the validity of the proposed hypotheses. The results of the study indicated that social media news overload has a significant effect on news avoidance, the need for news, and news filtering behavior. It was also observed that the need for news had a significant impact on news avoidance. In addition to this, it was also revealed that the need for news significantly mediated the relationship between social media news overload and news avoidance; however, it did not mediate the relationship between social media news overload and news filtering. Lastly, it was identified that media literacy significantly moderated the relationship between the need for news and news avoidance and it did not moderate the relationship between the need for news and news filtering behavior. This study has made important theoretical contributions by advancing the current literature in terms of the empirical evidence that indicates a significant relationship between social media news overload, news avoidance, and news filtering. Practically, this study contributed by emphasizing the need to encourage and train people to use strategies to seek relevant news in a vast repository of information available through information technology.

## Introduction

The popularity of social media has enabled the dissemination of news, not only rapidly but also widely, to the vast population (Sun et al., 2021). People gain information regarding current affairs and politics from social media rather than from mass media (Song et al., 2020). Nonetheless, energy, time, and effort are required to determine accurate and relevant news from excessive information provided through social media (Zhang et al., 2014). Moreover, repeated breaking of news also enhances the possibility of news overload for the people (Song et al., 2017). The present study intended to examine the role of social media news overload on social media news avoidance and filtering along with the mediating role of the need for news and moderation of media literacy. Theoretically, the study provides greater insights for the readers to understand the impact of social media news overload, because this topic is quite crucial in the present era of social media which is widely used among masses. Undoubtedly, need for news among the people might get affected when overloaded with social media news because too much information increases level of anxiety among people. The reader would also understand that media literacy plays a significant role in avoiding and filtering the available news on social media. Practically, the social media page owners would understand that overloading public with massive information would bring negative consequences such as low need for news, avoidance, and filtering of news. However, media literacy among masses can help to hamper the adverse effect of need for need on avoidance and filtering of social media.

Regular avoidance or refraining from social media news is known as “social media news avoidance” (Skovsgaard and Andersen, 2019). News avoidance can be either unintentional or intentional. Unintentional news avoidance happens when people use additional sources of content in social media rather than news while intentional news avoidance occurs when people start avoiding the news as it causes stress and anxiety among them (Karlsen et al., 2020). There are four approaches to news avoidance operationalization (Skovsgaard and Andersen, 2019). The first approach involves news exposure groups defined through clustering techniques. The second approach highlights the difference between news seekers and news avoiders based on the pattern of their news consumption (Aalberg et al., 2013). The third approach involves the consumption of news based on a specific time (e.g., fewer than 3 days per week or less than twice a month). Lastly, the fourth approach of news avoidance emphasizes categorizing the individuals as news avoiders. These various measures and definitions have enabled us to understand the dynamics of news avoidance among the masses.

Social media news filtering uses algorithmic filtering to display only such particular information to the users which are needed by the users (Goyanes et al., 2021b). These algorithms present the information for the users after its classification, association, and filtration of the information (Rader and Gray, 2015). According to Demirkan et al. (2016), algorithmic filtering provides news content that matches with the political orientation and preferences of the users, thus ensuring confirmation biases. The social media experiences of the users are usually shaped by these algorithms; however, the users are generally not aware of them. A study showed that 62.5% of Facebook users were unaware of the existence of such algorithms in Facebook’s news feed (Eslami et al., 2015). Moreover, social media companies use algorithms by an automated process, and the users are unable to control these algorithms. According to Goyanes et al. (2021b), the users who dislike an overload of information on social media require tools to filter out irrelevant information from their feed. Kapoor et al. (2017) stated that the users tend to rely on news articles shared by their friends on social media rather than from the news companies. This suggests that social media news filtering proves to be beneficial for the users; otherwise, it creates anxiety and unrest among the users (Leonard and Williams, 2021).

Various consequences are linked to information overloads like stress, anxiety, and loss of control (Diehl et al., 2015). Additionally, information overload tends to make it difficult for them to process the information, individuals tired, and difficult for them to determine appropriate information (Crook et al., 2015). Information overload includes news overload; however, these two are difficult to distinguish from each other. However, theoretically, information overload involves different kinds of information sources such as promotions, advertisements, and announcements, while news overload involves exposure to vast news (Roetzel, 2018). Moreover, information overload has a close relationship with cognitive psychology, and this has been extensively explored through empirical evidence in the fields of consumer behavior and marketing research. However, news overload has been recently investigated in the discipline of communication (Song et al., 2017). Furthermore, in the context of social media, news overload has made people utilize their energy and cognitive abilities because social media provides too much information to its users, hence making them exhausted and stressed out (Adhikari and Panda, 2019). A huge amount of information on social media tends to cross the cognitive limits of the users while evaluating and analyzing the news; therefore, the users feel overloaded and overwhelmed (Schmitt et al., 2017).

People around the globe are inclined toward the use of social media and they feel the need for news which has drastically increased among the masses (Ahmadi et al., 2021). The news consumption pattern of the young population has gradually shifted over the years. The users find social media news more authentic than traditional sources of news, and hence seek news from social media channels relatively more. In addition, individuals browse the Internet for information and news which is abundant and readily available, thus hardly relying on traditional mediums (Turcotte et al., 2015). Moreover, scholars have found that people nowadays acquire information from social media as it is less time-consuming, content is more interesting, and is highly preferred. In order to stay updated, individuals have become more prone to the use of social media for gathering information and news because they want to stay updated and share information with others. Further, people are becoming more aware of the authenticity and availability of information around them due to social media; therefore, the need for news among them has been increasing at a fast pace (Thompson et al., 2019). However, overload of news might affect their news consumption behavior because it becomes difficult to evaluate too much information; therefore, they avoid too much news or filter it out (Ku et al., 2019).

With too much information available over the Internet, media literacy among the people enables them to evaluate and analyze the information if the news is accurate and reliable (Tamplin et al., 2018). Individuals use their critical thinking skills to process high-quality news. Undoubtedly, individuals have to practically apply their knowledge about media news rather than just gaining it related to industries and current affairs; therefore, media literacy is crucial for the people. Moreover, media literacy is beneficial in understanding the essence of news messages and helps to avoid the circulation of fake news around media by avoiding or filtering it out. Individuals high in media literacy understand the purpose of news messages, evaluate the quality of news, differentiate opinions from facts, identify biases or favoritism, and share informed points on social media platforms (Ku et al., 2019). In addition, media literacy among the masses comes with time when individuals see a piece of particular information circulating social media in a different context; then, individuals can evaluate whether that information is reliable or not. Nonetheless, media literacy plays a crucial role in evaluating the news and understanding whether it should be shared with others or not (Cho et al., 2022).

The studies reviewed above have shown the consequences of social media news overload such as news avoidance, fatigue, anxiety, or news analysis paralysis (Bala et al., 2021). However, limited research has been carried out to examine the role of social media news overload on social media news avoidance and social media news filtering in a single framework. Moreover, Park (2019) suggested incorporating the need for news as a mediator in the relationship between social media news overload and social media news avoidance, and social media news filtering. Likewise, the moderating role of media literacy in the relationship between the need for news and social media news avoidance and social media news filtering has not been investigated earlier; thus, it is yet to be explored (Chen and Masullo Chen, 2019). Therefore, based on these research gaps, the present study intends to accomplish the research objectives which are to examine the effect of social media news overload on social media news avoidance, to analyze the effect of social media news overload on the need for news, to determine the role of social media news overload on social media news filtering, to understand the effect of need for news on social media news avoidance, to analyze the role of need for news on social media news filtering, to understand the mediating role of need for news in the relationship between social media news overload and social media news avoidance and in the relationship between social media news overload and social media news filtering, and to examine media literacy as a moderator in the relationship between need for news and social media news filtering.

## Review of Literature and Hypotheses Development

The present study examined the consumers of China to investigate the role of social media news overload on social media news avoidance and social media news filtering. The study also determined the mediating role of the need for news in the relationship between social media news overload and social media news avoidance and also in the relationship between social media news overload and social media news filtering. Moreover, the current study investigated the moderating role of media literacy in the relationship between social media news overload and social media news avoidance and also in the relationship between social media news overload and social media news filtering. The following theory was incorporated to support the framework of this study:

### New Media Theory

New media theory fosters the digital technologies implications, beginning from computer-mediated communication to provide socio-political configurations and leading toward the development of digital culture around the globe. Marshall McLuhan invented the new media theory in the early 1990s after the emergence of the World Wide Web. This theory has been divided into three sections based on themes that were developed throughout the evolution (An et al., 2021). These sections are identity, politics, and technology. Identity highlights the questions related to the relationship of subjectivity with digital media, specifically focusing on the new patterns of social interaction and identity formation that are linked with the new digital culture. Politics highlights the questions related to the role played by digital media in shaping the patterns of labor and taking possible political action. Technologies focus on media technologies and involve investigation on everyday engagements of people with mobile phone software and interfaces, to larger technological infrastructure.

New media theory has been rooted in the themes which involve two crucial conceptual traditions (Roetzel, 2018). First, critical theory and continental philosophy were the basis of theoretical work, and secondly, cultural studies were used to understand the dynamics of media studies to examine the questions related to value, representation, and agency. Moreover, the theory highlights vast information gained through the new media and the way the audience perceives it (Fischer and Reuber, 2011). The fast expansion of this field suggests that the bibliography is not exhausted but the problems and conceptualizations of this field are open for debate (Vallor, 2011). The new media theory suggests the significance of the latest media types in the current world and how important such media channels are for people and their work. Therefore, the present study incorporated new media theory for the theoretical support of the study model in terms of social media news perspective.

### Relationship Between Social Media News Overload and Social Media News Avoidance

The consequences of information overloading include stress, anxiety, and exhaustion which have been identified by researchers; hence, the need for social media news avoidance evolves (Wang et al., 2022). The individuals feel tired while processing and analyzing a large amount of information acquired from different sources to unearth relevant information (Goyanes et al., 2021a). Van Erkel and Van Aelst (2020) studied the outcome of excessive news on Americas, and they found that 70% of the Americas avoid news consumption when overloaded with the news. Similarly, another report on digital news by Tunney et al. (2021) showed that 57% of the worldwide population “often” avoids social media news subjected to social media news overload. Plass and Kalyuga (2019) stated that, based on the cognitive load theory, people have a certain bandwidth to take information and process it, while the rest of the information gets limited or blocked. Thus, too much information certainly leads to information avoidance. Furthermore, studies have been conducted in the past to examine the issue of social media news overload, but mainly the precursors such as the use of particular news platforms, news interest, consequences of news overload, or demographics have been analyzed (Song et al., 2017). However, the impact of social media news overload on social media news avoidance is in its infancy (Park, 2019), therefore to examine this relationship, the following hypothesis has been formulated:

*H1*: Social media news overload has an effect on social media news avoidance.

### Relationship Between Social Media News Overload and Need for News

Researchers have explored the outcomes of social media news overload such as the effect on mental health, tiredness among people, and loss of control (Ahmadi et al., 2021). However, the influence of social media news overload on the need for news has not been explored before. Some of the studies have indirectly investigated the antecedents of the need for news such as fake news Kumar et al. (2021) and news from brands (Chen and Cheng, 2020). Moreover, a recent study conducted by de Hoog and Verboon (2020) examined the role of daily news exposure and emotional state with the mediation of news-seeking behavior. This study had explicitly found that too much news exposure impacts the news-seeking behavior of the people along with their emotional state. This study could be indirectly related to the present study as an overload of information affects the need for news; however, the direct relationship of social media news overload with the need for news has not been explored. Therefore, to examine this relationship, the following hypothesis has been developed as:

*H2*: Social media news overload has an effect on need for news.

### Relationship Between Social Media News Overload and Social Media News Filtering

Apart from social media news avoidance, individuals use other techniques such as social media news filtering when encountered with news overload on social media (Van Erkel and Van Aelst, 2020). News consumers process news by filtering unimportant and irrelevant information from a large amount of information for disseminating it to the masses. Moreover, comparing social media news with traditional news media, social media news is filtered out using network-filtered or algorithm filters; however, traditional news media does not provide such facilities to its users (Guan et al., 2022). On the contrary, social media does not have the editorial team to provide information veracity and journalism quality. Lee et al. (2019) claimed that social media users come across a large amount of news from various sources with varying degrees of credibility. Thus, these users feel the need to minimize the cognitive burden of processing the news by using social filtering. According to Chen and Masullo Chen (2019), social media users can get credible news from abundant information by putting in less effort through the use of social filtering that provides selected and customized news according to the set filters. Although these studies have provided the association of news overload with news filtering, however Park (2019) posited that there is still room to investigate the impact of social media news overload on social media news filtering; thus, filling this gap in the literature, the following hypothesis has been posed as:

*H3*: Social media news overload has an effect on social media news filtering.

### Relationship Between Need for News and Social Media News Avoidance

The individual’s need for news arises when exposed to news in a technologically intensive environment which enables them to gain as much knowledge as possible (Toff and Kalogeropoulos, 2020). Social media users tend to seek news to share it with their friends and colleagues since this is possible only when they are well-informed and updated (Song et al., 2020). The users gain more knowledge through social media as compared to traditional news media; hence, their need for news is fulfilled through social media. Theoretically, individuals with a higher need for news may experience unpleasant news on social media that might influence them negatively; hence, they indulge in social media news avoidance behaviors (Goyanes et al., 2021a). These users are less exposed to traditional news channels; therefore, they are unable to fully understand the news content (Momsen and Ohndorf, 2022). Additionally, social media users high in need for news merely glance over the headlines and move to the next post without understanding the significance or essence of the news (Toff and Palmer, 2018). Also, most of the time, the need for news for such users becomes frustrating as they find it difficult to comprehend the news; thus, they start to avoid the news. According to Park (2019), the need for news is an important topic of discussion in the context of social media news and its outcomes are yet to be studied. Also, the empirical evidence of the significance of the need for news on social media news avoidance is limited in the literature; therefore, the following hypothesis has been posed as:

*H4*: Need for news has an effect on social media news avoidance.

### Relationship Between Need for News and Social Media News Filtering

The emergence of social media has provided an opportunity for people to gain news from multiple sources which has increased their need for news (Gordon et al., 2022). Social media users rely on different sources but with the ample information over the Internet, they are unable to evaluate the credibility and quality of the information. Thus, this gives rise to social media news filtering (Collins et al., 2020). Moreover, a study conducted by web censorship has found that people exhibiting news-seeking behavior tend to use news filtering on social media. The reason behind such behaviors was that the users need relevant news and want to filter out the irrelevant ones. Ahmed (2021) claimed that the major shift and increase in encrypted traffic indicates that users directly access the news using censorship-bypass mechanisms because their need for news allows them to indulge in news filtering methods. Another study revealed that 75 percent of social media users encounter a blocked page for YouTube while performing a search engine query (Swart, 2021). Various degrees of Internet filtering prevail in different regions, but surprisingly little research has been conducted on this subject (Jamali and Shahbaztabar, 2017). Thus, this calls for investigating the precursors and antecedents of news filtering. Moreover, Park (2019) suggested exploring the role of the need for news in the context of social media. Therefore, to address these gaps, the study proposed the following hypothesis:

*H5*: Need for news has an effect on social media filtering.

### Need for News as a Mediator

Access to social media is simple for the users; therefore, users tend to upload and share almost all the information either relevant or irrelevant (Kim et al., 2020), thus promoting social media news overload. This increase in news overload fosters the need for news among social media users (Jamali and Shahbaztabar, 2017). Moreover, the users need reliable and credible information; hence, they either avoid news on social media or filter it (Ahmadi et al., 2021). A study undertaken by Goyanes et al. (2021b) found that news overload increases news exposure which develops the need for news among the users, but too much news exposure creates frustration, anxiety, tiredness, and loss of control. Such reactions damage the mental health of the users; hence, they prefer to avoid social media news or filter the news.

Furthermore, information technologies allow the processing of information, but technology is deemed to be contributing to news or information overload because it enhances the amount of news that people acquire (Ku et al., 2019). When a large amount of information is present, people cannot process additional information considering their information-processing capacity; therefore, it results in poor ability to process information and psychological dysfunction (Thompson et al., 2019). This ultimately brings in the fact that people start avoiding and filtering social media news. The role of the need for news as a mediator has not been investigated before. Also, Park (2019) suggested examining the need for news as a mediator in the relationship between social media news overload and social media news avoidance and the relationship between social media news overload and social media news filtering. Thus, to address the gap in the literature, the following hypothesis has been posed as:

*H6*: Need for news mediates the relationship between social media news overload and social media news avoidance.

*H7*: Need for news mediates the relationship between social media news overload and social media news filtering.

### Media Literacy as a Moderator

The need for news has been increasing with the proliferation of social media which points out the fact that the role of media literacy cannot be denied (Vesali et al., 2022). Individuals high in media literacy have the ability to acquire relevant information from pool information (Vraga and Tully, 2019). The critical thinking abilities of such individuals are high which makes them eliminate unreliable and incredible information. Moreover, social media users who understand and comprehend the news acquired from various sources can easily avoid or filter irrelevant news from social media. According to (Tamplin et al. (2018)), media literacy plays an important role in shared content because people high in media literacy know what to share with the people and avoid sharing misinformation. A study by Ku et al. (2019) was conducted on adolescents to examine the impact of social media literacy on news-seeking motivation. The result of the study showed that media literacy has a positive impact on news-seeking motivation, indicating that people high in media literacy tend to have increased news-seeking motivation. Moreover, the role of media literacy as a moderator has not been explored in the information technology literature before; therefore, it calls for investigating the moderating role of media literacy. Also, Chen and Cheng (2020) suggested incorporating media literacy as a moderator in the relationship between the need for news and social media news avoidance and the relationship between the need for news and social media news filtering. Thus, the following hypothesis has been posited to address the gap:

*H8*: Media literacy moderates the relationship between need for news and social media news avoidance.

*H9*: Media literacy moderates the relationship between need for news and social media news filtering.

**Figure 1:**
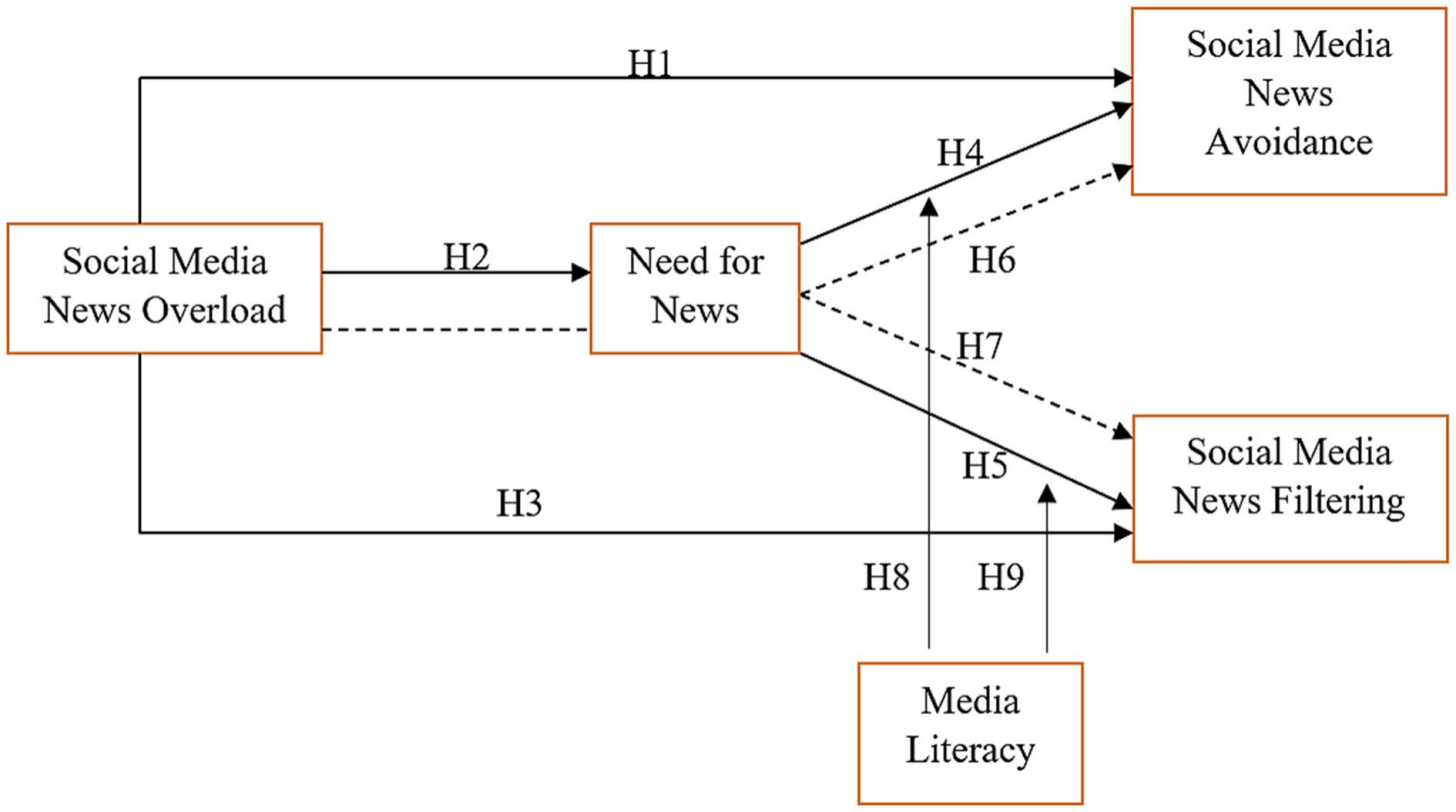
Theoretical framework.

The conceptual framework has been established based on the suggested literature and hypothesis as show in Figure 1.

## Methodology

The impact of independent variables on dependent variables was examined through the study’s hypotheses. The hypotheses of the study were validated with the help of a deductive approach and based on this approach, a quantitative research design was adopted. This research design was useful in eliminating the biases from the study. A self-administered survey was developed for data collection. The participants were told to be as natural as possible in order to obtain accurate results. The data were collected from the social media users through the use of a convenience sampling technique. According to Nawaz et al. (2019), convenience sampling helps to gather data in a fast and convenient manner from readily available participants. A total of 420 questionnaires were disseminated to the study participants out of which 358 questionnaires were analyzed for conducting the study. The author took 3 weeks to acquire the responses and screened them. During the screening process, 62 responses were discarded as they were either incomplete or improper; thus, the response rate obtained was 85.2%. The determined sample size was 358 and the unit of analysis was social media users of China.

### Statistical Tool

The structural equation modeling (SEM) technique was deployed for the present study; therefore, Smart-PLS 3.3.3 software was used to analyze the data. This software helps to thoroughly provide insights into the data by developing a path model in a very short period (Avotra et al., 2021; Xiaolong et al., 2021). Smart-PLS uses a measurement model and structural model for examining the data. Firstly, the measurement model provides data validity including AVE, factor loading, VIF, Fornell and Larker Criterion, and Heterotrait-Monotrait ratio (Nawaz et al., 2020). The measurement model also helps in determining data reliability using Cronbach alpha and composite reliabilities. Moreover, the software then examines the data using a structural model once the obtained values for data validity and reliability are within the threshold level. In this second stage, the acceptance or rejection of the hypotheses is determined through *p* values, t-statistics, and *F*-values.

### Measurement

A 5-point Likert scale was deployed to acquire the responses for each item of the variable (i.e., social media news overload, social media news avoidance, social media news filtering, need for news, and media literacy). The measurement for each variable of the study has been provided below:

#### Social Media News Overload

A 3-items scale for social media news overload was adopted from Song et al. (2017).

#### Social Media News Avoidance

A 3-items scale for social media news avoidance was adopted from Pentina and Tarafdar (2014).

#### Social Media News Filtering

A 2-items scale for social media news filtering was adopted from Park (2019).

#### Need for News

A 4-items scale for the need for news was adopted from Gil de Zúñiga et al. (2017).

#### Media Literacy

The 4-items scale for media literacy was adopted from Ashley et al. (2013).

### Demographic Profile

The analysis of the demographic characteristics of the respondents depicts (see Table 1) that there were 213 males and 145 females who participated in this study. The male and female participation stood at 59.50 and 40.50%, respectively. It can also be observed that 62.01% of the respondents were aged between 20 and 30 years, 27.37% were 31–40 years of age, and 5.59% were aged between 41 and 50 years, whereas, 5.03% were above the age of 50 years. Furthermore, it can also be observed that 55.03% of the respondents possessed a Bachelor’s education, 35.47% had done a Master’s while 9.50% had obtained a Ph.D. or some other qualification.

**Table 1:** Demographics analysis.

**Demographics**	**Frequency**	**Percentage**
**Gender**		
Male	213	59.50%
Female	145	40.50%
**Age (years)**		
20–30	222	62.01%
31–40	98	27.37%
41–50	20	5.59%
Above 50	18	5.03%
**Education**		
Bachelors	197	55.03%
Masters	127	35.47%
Ph.D. and others	34	9.50%

*N = 358.*

## Data Analysis and Results

### Measurement Model

**Figure 2:**
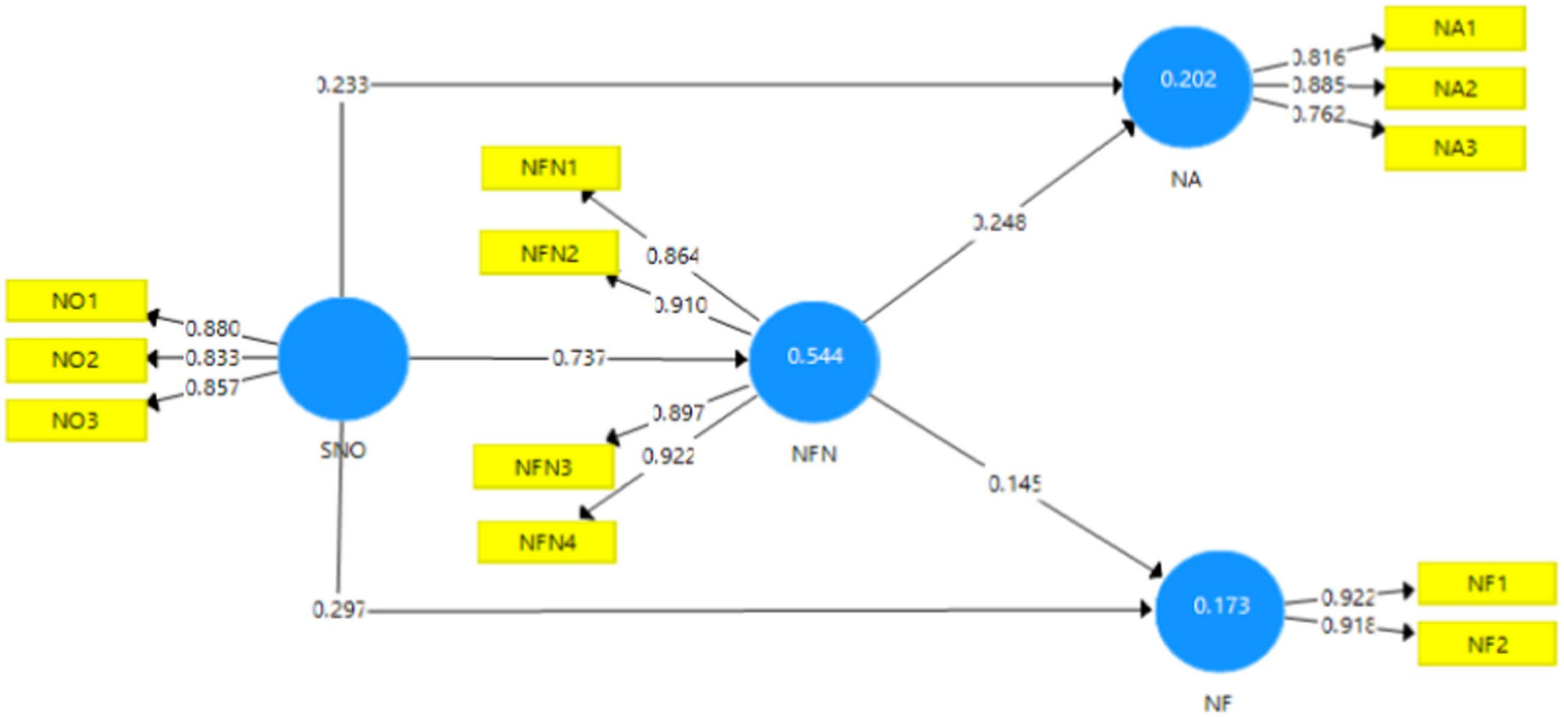
Output of measurement model without moderation. SMO, Social Media Overload; NA, News Avoidance; NF, News Filtering; NFN, Need for News.

**Figure 3:**
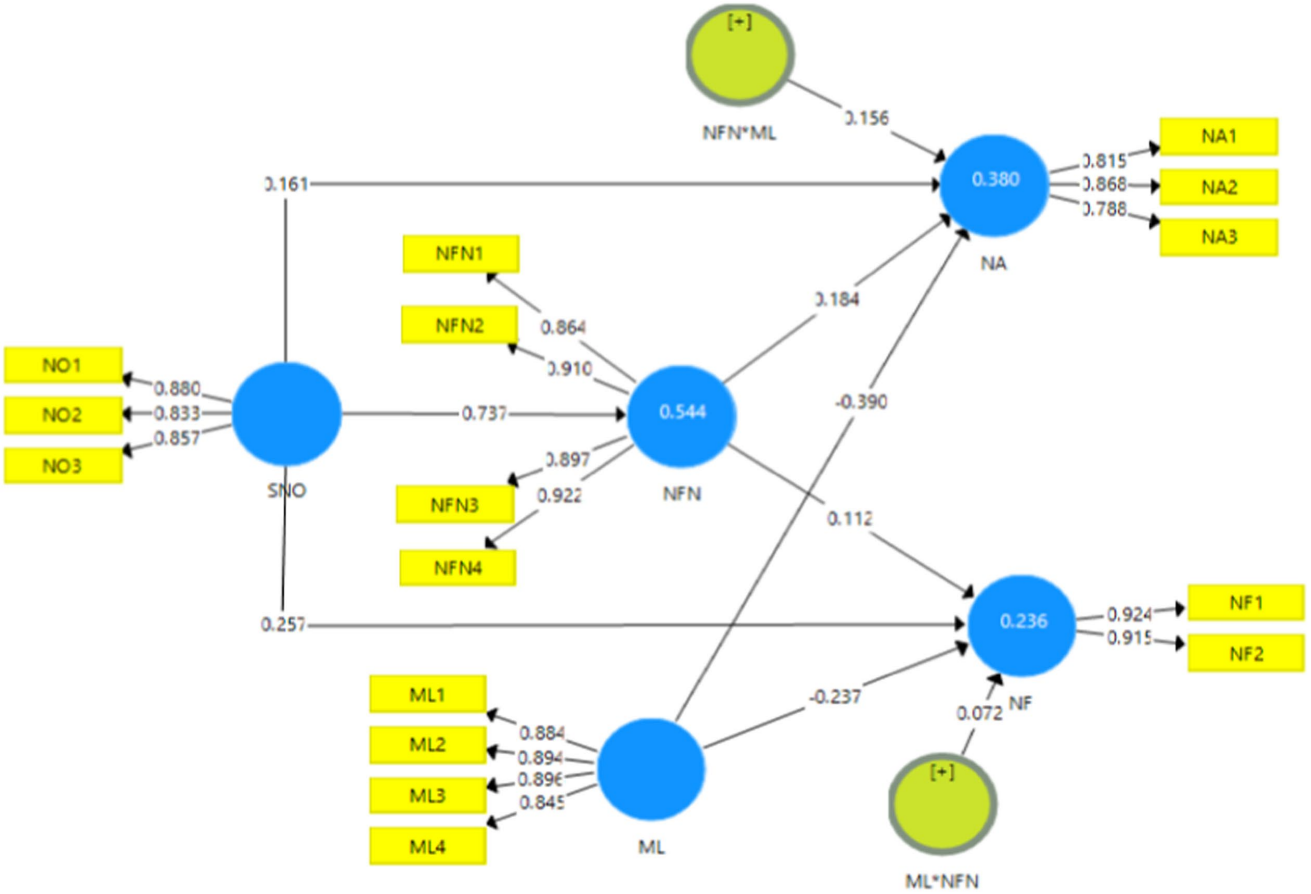
Output of measurement model with moderation. SMO, Social Media Overload; NA, News Avoidance; NF, News Filtering; NFN, Need for News; and ML, Media Literacy.

The figures are given below, i.e., Figures 2, 3 denote the outcome of the measurement model without and with moderation. These figures depict the extent to which the predictor variables contribute or have an effect on the outcome variable that is being analyzed in this study.

Table 2 demonstrates the factor loading along with the VIF values obtained against each item of social media news overload, social media news avoidance, social media news filtering, and need for news. As per Jordan and Spiess (2019), the acceptable values of factor loadings should be higher than 0.60. The factor loadings for obtained against each item for the current study ranged from 0.762 to 0.922. Hence, it can be ascertained that all values of factor loadings were fair. Moreover, the variance inflation factor (VIF) is a measure of determining collinearity in the model. According to Henseler et al. (2015), the acceptable values of VIF should be higher than 5. The VIF values that were obtained against each item of this study ranged from 1.505 to 4.600 on the basis of which it can be ascertained that collinearity was not an issue in this study.

**Table 2:** Model assessment (direct model).

	**Factor Loadings**		**VIF**	**Construct reliability and validity**		
				**α**	**Composite Reliability**	**AVE**
Social Media News Overload	SMO1	0.880	2.057			
	SMO2	0.833	1.863	0.820	0.892	0.734
	SMO3	0.857	1.691			
Social Media News Avoidance	NA1	0.816	1.505			
	NA2	0.885	1.681	0.766	0.862	0.676
	NA3	0.762	1.530			
Social Media News Filtering	NF1	0.922	1.920	0.818	0.917	0.846
	NF2	0.918	1.920			
Need for News	NFN1	0.864	2.586			
	NFN2	0.910	4.106	0.920	0.944	0.807
	NFN3	0.897	3.092			
	NFN4	0.922	4.600			

SMO, Social Media Overload; NA, News Avoidance; NF, News Filtering; NFN, Need for News; VIF, Variance Inflation Factor; *α*, Cronbach Alpha; and AVE, Average Variance Extracted.

Moreover, the construct reliabilities and validities have also been depicted in the table shown below. The construct reliability and validity were measured using Cronbach’s alpha (α), composite reliability, and average variance extracted (AVE). If the Cronbach value is higher than 0.70, the construct is deemed as reliable. Moreover, the acceptable threshold for composite reliability must also be greater than 0.70 (Peterson and Kim, 2013). It can be observed that all values of Cronbach alpha and composite reliability successfully met these threshold levels. Hence, it can be concluded that the constructs were reliable. Moreover, Benitez et al. (2020) suggested that the acceptable threshold for AVE should be higher than 0.60. It can be observed from Table 2 that all values of AVE fulfilled this criterion thus indicating the presence of convergent validity.

The presence of discriminant validity was checked through the conductance of the HTMT ratio and the Fornell and Larcker test. The results can be seen in Table 3. Discriminant validity determines whether or not one construct is unique and different from another construct. The HTMT values shown in the table are all below 0.90 which suggests the presence of discriminant validity (Benitez et al., 2020). Furthermore, the values on the top of the column in the Fornell Larcker criteria must be higher than the values that are below them (Franke and Sarstedt, 2019). It can be observed that all values at the top of each column in the Fornell Larcker criterion fulfilled this assumption. Therefore, it can be concluded that discriminant validity was present among the constructs of this study.

**Table 3:** Discriminant validity.

**Fornell-Larcker criterion**					**Heterotrait-Monotrait ratio**				
Constructs	**NA**	**NF**	**NFN**	**SMO**	**Constructs**	**NA**	**NF**	**NFN**	**SNO**
**NA**	0.822				**NA**				
**NF**	0.526	0.920			**NF**	0.659			
**NFN**	0.421	0.364	0.898		**NFN**	0.481	0.418		
**SMO**	0.417	0.404	0.737	0.857	**SNO**	0.505	0.492	0.838	

SMO, Social Media Overload; NA, News Avoidance; NF, News Filtering; and NFN, Need for News.

Table 4 given below exhibits the R-square values that were obtained for news avoidance, news filtering, and need for news. According to Sarstedt et al. (2014), the R-square denotes the sustainability of the model and the acceptable threshold for R-square should be close to 0.50. The R-square values obtained against news avoidance, news filtering, and need for news were 0.202, 0.173, and 0.544 which indicate that the proposed model is sustainable. Moreover, the cross-validated redundancy is indicated through the Q-square values. According to Shiau et al. (2019), the Q-square value should be higher than zero. It can be observed that all values of Q-square successfully fulfilled this assumption. Therefore, the fitness and sustainability of the model have been established.

**Table 4:** R-Square values and Q-Square values for the variables.

	**R-Square**	**Q-Square**
NA	0.202	0.122
NF	0.173	0.133
NFN	0.544	0.412

NA, News Avoidance; NF, News Filtering; and NFN, Need for News.

Table 5 demonstrates the inner-VIF values that depict the collinearity within the proposed model. As per Sarstedt et al. (2014), the inner variance inflation factor (VIF) values should be less than 5. It can be seen from the table that all inner-VIF values were below 5 which suggests that collinearity was not present between the constructs of the study.

**Table 5:** Collinearity statistics (inner-VIF values).

	**ML**	**ML** ^*^ **NFN**	**NA**	**NF**	**NFN**	**NFN** ^*^ **ML**
ML			1.068	1.069		
ML^*^NFN				1.049		
NA						
NF						
NFN			2.212	2.214		
NFN^*^ML			1.046			
SNO			2.219	2.218	1.000	

N = 358, SMO, Social Media Overload; NA, News Avoidance; NF, News Filtering; NFN, Need for News; and ML, Media Literacy.

### Structural Model

**Figure 4:**
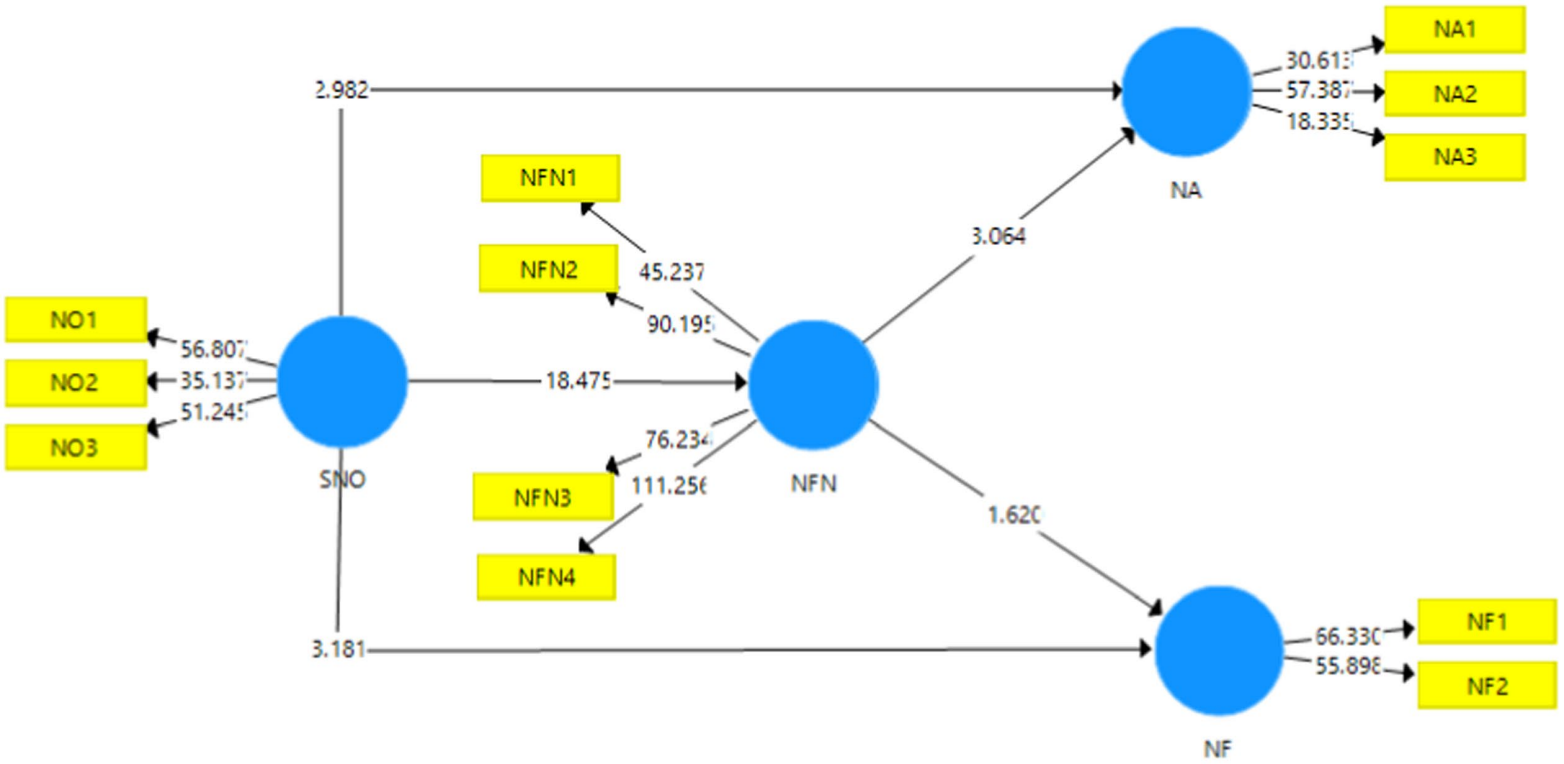
Structural model bootstrapping without moderation. SMO, Social Media Overload; NA, News Avoidance; NF, News Filtering; NFN, Need for News.

Figure 4 shown below indicates the outcome of the structural model bootstrapping that was undertaken without the effect of moderation. The t-statistic values have also been given. The validity of the hypotheses is determined through the PLS-SEM bootstrapping model that is undertaken at a 95% confidence interval.

Tables 6, 7, and 9 depict the outcome of the direct, indirect, and moderating effects that were undertaken in order to ascertain the validity of the proposed hypotheses. The acceptance and rejection of the hypothesis depend upon the t-statistic and value of *p*. Johnson (2019) suggests that the value of the t-statistic should be higher than 1.96, whereas Di Leo and Sardanelli (2020) posit that the acceptable threshold for value of *p* should be less than 0.05. In addition to this, the effect sizes are also indicated through the *f* ^2^ values which denote the strength of the proposed model. According to Meng and Bari (2019), the value of *f* ^2^ near to 1 indicates higher strength, whereas those near to 0 indicate lower strength.

**Table 6:** Direct effects of the variable.

Paths	H	O	M	SD	T-statistics	Effect Size (*f*^2^)	Value of *p*	Results
SMO➔NA	H1	0.233	0.237	0.078	2.982	0.031	0.003	Accepted
SMO➔NFN	H2	0.737	0.738	0.040	18.475	1.191	0.000	Accepted
SMO➔NF	H3	0.297	0.291	0.093	3.181	0.049	0.002	Accepted
NFN ➔NA	H4	0.248	0.250	0.081	3.064	0.035	0.002	Accepted
NFN➔NF	H5	0.145	0.150	0.090	1.620	0.012	0.106	Rejected

H, Hypothesis; O, Original Sample; M, Sample Mean; SD, Standard Deviation; SRMR = 0.076, NFI = 0.752, SMO, Social Media Overload; NA, News Avoidance; NF, News Filtering; NFN, and Need for News.

**Table 7:** Indirect effects of the variable.

**Paths**	**H**	**O**	**M**	**SD**	**t-statistics**	**Value of *p***	**Results**
SMO ➔ NFN ➔ NA	H6	0.183	0.183	0.059	3.125	0.002	Accepted
SMO ➔ NFN ➔ NF	H7	0.107	0.112	0.068	1.565	0.118	Rejected

H, Hypothesis; O, Original Sample; M, Sample Mean; SD, Standard Deviation; SMO, Social Media Overload; NA, News Avoidance; NF, News Filtering; and NFN, Need for News.

The table given above depicts the results of the testing of the hypothesis, H1, H2, H3, H4, and H5. H1 predicted that social media overload (SMO) had an effect on news avoidance (NA). The t-statistic and values of *p* are *t* = 2.982 and *p* = 0.003 which mean that this hypothesis has been accepted. The value of *f* ^2^ is 0.031 which indicates that the model strength is very low. H2 proposed that social media overload (SMO) had an effect on the need for news (NFN). This hypothesis was also accepted as indicated by the t and values of p which are 18.475 and 0.000, respectively. The *f* ^2^ is 1.191 which suggests that the model strength is very strong. The third hypothesis, H3 predicted that social media overload (SMO) had an effect on news filtering (NF). The t value is 3.181 and the value of p is 0.002 which means that this hypothesis has also been accepted. The effect size is 0.049 which means that the model strength was weak. The fourth hypothesis, H4 posited that the need for news (NFN) had an effect on news avoidance (NA). This hypothesis is also accepted as indicated by the t and values of p that are 3.064 and 0.002, respectively. The effect size (*f* ^2^ = 0.035) indicates that the model strength was weak. The fifth hypothesis, H5 proposed that the need for news (NFN) had an effect on news filtering (NF). This hypothesis was rejected because the t value was less than 1.96, i.e., 1.620 and the value of p was higher than 0.05, i.e., 0.106.

The value of the normed fixed index (NFI) is used to determine the fitness of the model. According to Elsayed and Aneis (2021), the values of NFI must range between 0 and 1. The value of NFI as shown in Table 6 came out to be 0.752 which suggests a high model fitness. The value of the standardized root mean square residual (SRMR) is 0.076 which is within the threshold range of 0–0.08 prescribed by Pavlov et al. (2020). This also indicates that the overall model was fit for the data.

Table 7 depicts the indirect effects of the constructs. The sixth hypothesis, H6 predicted that the need for news (NFN) mediated the relationship between social media overload (SMO) and news avoidance (NA). The values of *t* and *p* are 3.125 and 0.002 which suggest that this hypothesis has been accepted. The seventh hypothesis, H7 proposed that NFN mediated the relationship between SMO and news filtering (NF). This hypothesis has been rejected because the t value is below 1.96 and the value of p is above 0.05, i.e., 1.565 and 0.118, respectively.

**Figure 5:**
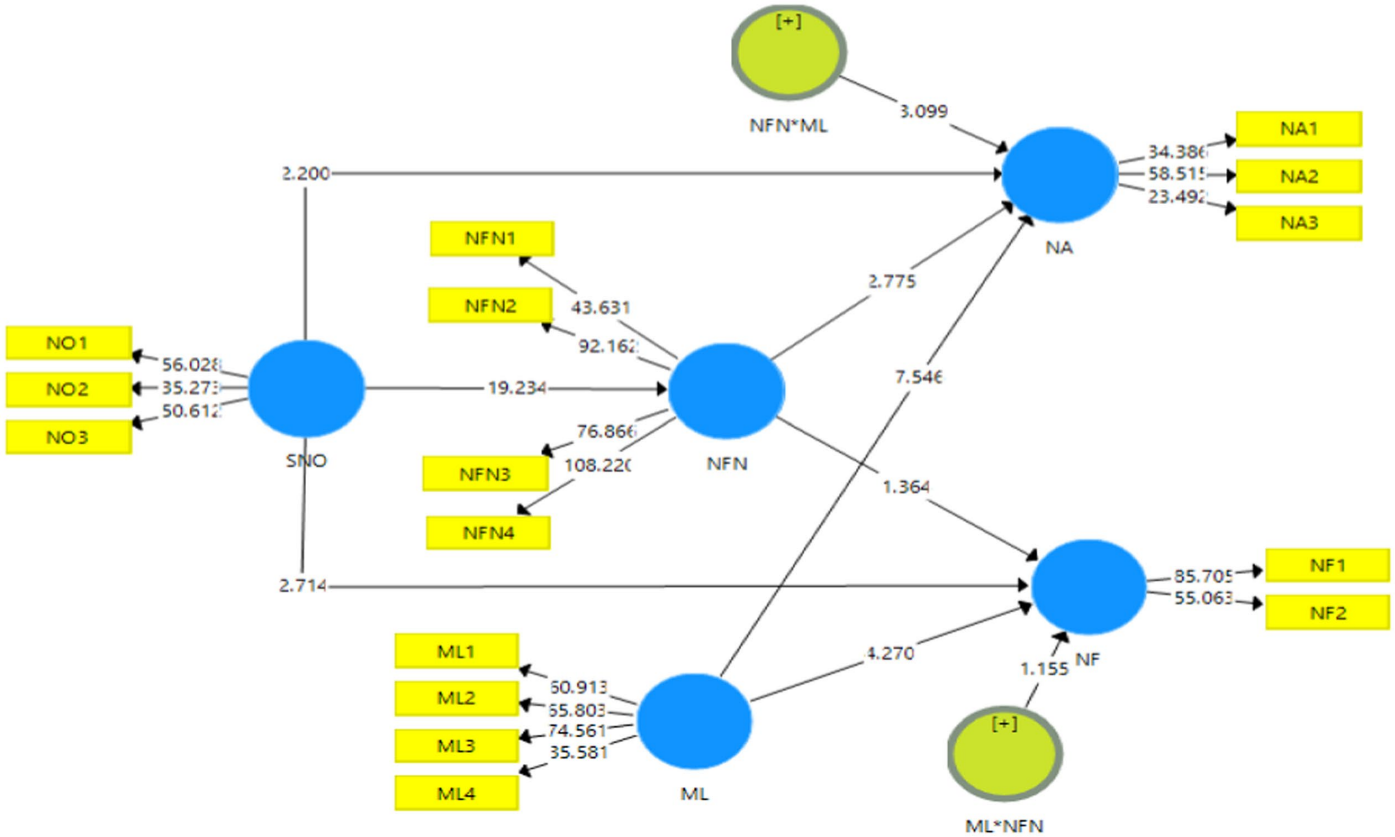
Structural model bootstrapping with moderation. SMO, Social Media Overload; NA, News Avoidance; NF, News Filtering; NFN, Need for News; and ML, Media Literacy.

The validation of the data was again examined this time in the presence of the moderating variable, i.e., media literacy (ML) as shown in Table 8. The measures of factor loadings, VIF values, Cronbach alpha, composite reliability, and AVE were used to examine the validity of the data in the presence of the moderator. It can be observed that all values of the above indicators surpassed the minimum threshold limits (Figure 5).

**Table 8:** Model assessment (moderation).

	**Factor Loadings**		**VIF**	**Construct reliability and validity**		
				**α**	**Composite Reliability**	**AVE**
Social Media News Overload	SMO1	0.880	2.057			
	SMO2	0.833	1.863	0.820	0.892	0.734
	SMO3	0.857	1.691			
Social Media News Avoidance	NA1	0.816	1.505			
	NA2	0.885	1.681	0.766	0.862	0.676
	NA3	0.762	1.530			
Social Media News Filtering	NF1	0.922	1.920	0.818	0.917	0.846
	NF2	0.918	1.920			
Need for News	NFN1	0.864	2.586			
	NFN2	0.910	4.106	0.920	0.944	0.807
	NFN3	0.897	3.092			
	NFN4	0.922	4.600			
Media Literacy	ML1	0.884	2.775			
	ML2	0.894	3.484	0.964	0.967	0.650
	ML3	0.868	3.054			
	ML4	0.845	2.959			

SMO, Social Media Overload; NA, News Avoidance; NF, News Filtering; NFN, Need for News; ML, Media Literacy; VIF, Variance Inflation Factor; *α*, Cronbach Alpha; and AVE, Average Variance Extracted.

Table 9 demonstrates the moderating effects of media literacy. The eighth hypothesis, H8 which predicted that media literacy (ML) moderated the relationship between the need for news and news avoidance has been accepted as indicated by the t and *p* values which are 3.099 and 0.002, respectively. However, the ninth hypothesis, H9 has been rejected because the value of *t* was less than 1.96, i.e., 1.155 and the value of *p* was higher than 0.05, i.e., 0.248. Hence, it can be concluded that media literacy does not moderate the relationship between the need for news and news filtering.

**Table 9:** Moderating effects of the variable.

**Paths**	**H**	**O**	**M**	**SD**	**t-statistics**	**Value of *p***	**Results**
NFN × ML ➔ NA	H8	0.156	0.160	0.050	3.099	0.002	Accepted
ML × NFN ➔ NF	H9	0.072	0.092	0.063	1.155	0.248	Rejected

H, Hypothesis; O, Original Sample; M, Sample Mean; SD, Standard Deviation; NA, News Avoidance; NF, News Filtering; NFN, Need for News; and ML, Media Literacy.

## Discussion

The gaps in the literature related to social media were addressed in the present study by taking data from social media users. The direct relationships of the study were initially examined, i.e., the impact of social media news overload on social media news avoidance, need for news, and social media news filtering. The direct relationships were also investigated in terms of examining the impact of the need for news on social media news avoidance and social media news filtering. The role of the need for news as a mediator in the relationship between social media news overload and social media news avoidance and social media news filtering was also investigated in the present study. Moreover, the study also evaluated the moderating role of media literacy in the relationship between the need for news and social media news avoidance and social media news filtering. The result of the study provides some useful contributions in the context of social media news and information. This study would help provide concrete recommendations for social media companies.

The first direct relationship or first hypothesis of the study was accepted indicating that social media news overload has an effect on social media news avoidance among social media users. These results were in harmony with the findings of the study conducted by Tunney et al. (2021). The reason could be that people get so frustrated and experience a higher cognitive burden from the overload of news that they want to avoid it. The second hypothesis of the study was accepted indicating that social media news overload affects the need for news among social media users. This could be because individuals get exposed to social media news overload which affects their need for news behavior. These findings were similar to the findings obtained by de Hoog and Verboon (2020). The third hypothesis of the study was accepted indicating that social media news overload has an effect on social media news filtering among social media users. Social media news overload affects the mental health of the users; hence, they feel to filter out most of the irrelevant news. These findings were in line with the results of the study carried out by Lee et al. (2019).

The fourth hypothesis of the study was accepted indicating that the need for news has an effect on social media news avoidance among social media users. The plausible reason could be a higher need for news develops social media news avoidance behavior among people because they require only reliable and credible news. Goyanes et al. (2021a) found similar results. However, the fifth hypothesis of the study was rejected indicating that need for news has no effect on social media news filtering among social media users. These results contradicted the findings of Collins et al. (2020). The reason for such results could be that although users need news to stay updated and tend to filter out the irrelevant news but the at times, they are exhausted in the process of filtering out the news; therefore, they abandon the process of seeking the news.

The sixth hypothesis of the study was accepted indicating that need for news mediates the relationship between social media news overload and social media news avoidance among social media users. Higher social media news overload creates higher news exposure among people which affects the need for news which ultimately enables them to avoid irrelevant news on social media. Similar findings were obtained by Goyanes et al. (2021b). The seventh hypothesis of the study was rejected indicating that need for news did not mediate the relationship between social media news overload and social media news filtering among social media users. These results contradicted the findings of Ku et al. (2019). One of the reasons could be users getting exhausted from too much information that they just stop the filtering out process.

The eighth hypothesis of the study was accepted indicating that media literacy moderates the relationship between the need for news and social media news avoidance among social media users. Media literacy plays a crucial role in understating, comprehending, and differentiating between reliable and unreliable news; thus, media literacy can regulate the effect of the need for news on social media news avoidance; therefore, the relationship came out to be true. These results were in harmony with the findings of the study conducted by Tamplin et al. (2018). The ninth hypothesis of the study was rejected indicating that media literacy does not moderate the relationship between the need for news and social media news filtering among social media users. These results contradicted the findings of Vesali et al. (2022). The plausible reason could be that users stop filtering news on social media because of the high cognitive burden, therefore, media literacy among the users cannot play its role.

## Theoretical and Practical Implications, Limitations and Future Directions, and Conclusion

### Theoretical Implications

The current study encompasses some important theoretical implications. Firstly, this study greatly advances the current literature by examining the impact of social media news overload on news avoidance, the need for news, and news filtering behavior. By doing so, this study has determined that social media news overload will drive people to avoid news seeking. Moreover, it has also been established that news overload will also compel people to engage in news filtering behavior under which they will start to filter out the news to seek relevant information. Another valuable contribution that was made by this study was that social media news overload also led to an increase in the need for news as people start to navigate for relevant information from a vast repository of news.

In addition to this, the present study also contributed to the existing body of knowledge by examining the mediating mechanism of need for news in the relationship between social media news overload, news avoidance, and news filtering behavior. In this way, this study advanced the current literature by establishing the mediating role played by the need for news in the relationship between news overload and news avoidance. On the other hand, the present study also investigates the moderating mechanism of media literacy in the relationship between the need for news, news avoidance, and news filtering behavior. By undertaking this investigation, this study had provided great insight by establishing the fact that media literacy buffers the relationship between the need for news and news avoidance.

### Practical Implications

This study also has some valuable practical implications. First of all, the usage of news has a strong and significant relationship with an increase in overall knowledge, awareness, and literacy as indicated in the study. Therefore, it is evident that if people perceive they are being overloaded with the news, they will either avoid looking for news or they will prefer filtering the relevant news from lots of news available. This might lead the users to the filtering of useful or relevant news and increases the chances of people getting exposed to irrelevant and invaluable information which in turn might cause a decrease in overall awareness and literacy. Therefore, it becomes imperative for the social media companies, government, and the people associated with the education and literacy awareness sector to encourage and educate people to exert efforts and apply strategies to find relevant news from a large repository of information. Furthermore, this allows the media agencies to provide valuable and relevant news to their audience to avoid bombarding them with news overload and consequently causing a decrease in their target audience. By undertaking such measures, the problem of news overload discouraging people to seek news can be mitigated.

### Limitations and Directions for Future Studies

Certain limitations were associated with the present study. The generalizability of the findings can be improved by broadening the sample size to acquire data from more respondents. This study can be replicated in other cultural contexts to gain a deeper insight into how news overload influences news avoidance and filtering behavior. This study had a cross-sectional design; hence, future studies can adopt a longitudinal approach by collecting data at various time intervals to improve the reliability of the results. Furthermore, this study only analyzed the mediating and moderating mechanisms of need for news and media literacy in the relationship between news overload, need for news, news avoidance, and news filtering behavior. Future studies can introduce other possible mediating and moderating mechanisms such as news efficacy, news handling, and social media knowledge in order to broaden the understanding regarding the influence of news overload on news avoidance and news filtering.

### Conclusion

The need to seek news and relevant information has become all the more important in this age as it significantly aids an individual in broadening their knowledge and awareness, and helping them in making better and informed decisions. However, if people are bombarded with excessive news, they might avoid seeking news or they might get indulged in the exhausting process of filtering out relevant news. Keeping this in mind, the present study undertook an inquiry to examine and investigate the impact of social media news overload on news avoidance and news filtering behavior through the mediating and moderating mechanisms of need for news and media literacy. For this purpose, Chinese social media users were chosen as respondents. The results reveal that social media news overload has a significant effect on news avoidance, need for news, and news filtering behavior. Moreover, it was also observed that need for news had a significant relationship with news avoidance. Furthermore, it was seen that need for news did not have an effect on news filtering. In addition to this, it was also revealed that need for news mediated the relationship between social media news overload and news avoidance, whereas it did not mediate the relationship between social media news overload and news filtering. Lastly, it was observed that media literacy moderated the relationship between need for news and news avoidance and it did not moderate the relationship between need for news and news filtering behavior.

## Data Availability Statement

The original contributions presented in the study are included in the article/Supplementary Material, and further inquiries can be directed to the corresponding author.

## Ethics Statement

The studies involving human participants were reviewed and approved by the South China Agricultural University, China. The patients/participants provided their written informed consent to participate in this study. The study was conducted in accordance with the Declaration of Helsinki.

## Author Contributions

The author confirms being the sole contributor of this work and has approved it for publication.

## Conflict of Interest

The author declares that the research was conducted in the absence of any commercial or financial relationships that could be construed as a potential conflict of interest.

## Publisher’s Note

All claims expressed in this article are solely those of the authors and do not necessarily represent those of their affiliated organizations, or those of the publisher, the editors and the reviewers. Any product that may be evaluated in this article, or claim that may be made by its manufacturer, is not guaranteed or endorsed by the publisher.
